# Aggressive Deeskalation der Strahlentherapie bei HPV-assoziierten Oropharynxkarzinomen auf der Basis der Hypoxiedynamik

**DOI:** 10.1007/s00066-021-01765-6

**Published:** 2021-03-25

**Authors:** Alexander Rühle, Nils H. Nicolay

**Affiliations:** 1 Klinik für Strahlenheilkunde, Universitätsklinikum Freiburg, Robert-Koch-Str. 3, 79106 Freiburg, Deutschland; 2 Deutsches Konsortium für Translationale Krebsforschung (DKTK), Partnerstandort Freiburg, Deutsches Krebsforschungszentrum (dkfz), Heidelberg, Deutschland

## Hintergrund und Ziel der Arbeit

Patienten mit HPV-positiven Oropharynxkarzinomen weisen eine sehr gute Prognose nach Radiochemotherapie (RCT) auf, leiden jedoch häufig und längerfristig unter therapiebedingten Toxizitäten. Unabhängig vom HPV-Status konnte in der Vergangenheit gezeigt werden, dass eine fehlende prätherapeutische Tumorhypoxie oder ein rasches Auflösen der Tumorhypoxie innerhalb der ersten 10 Bestrahlungsfraktionen bei Plattenepithelkarzinomen der Kopf-Hals-Region mit einer signifikant verbesserten Prognose einhergeht. Die Autoren der vorliegenden Studie postulieren nun, dass eine auf funktioneller Hypoxiebildgebung basierende aggressive Deeskalation der RCT bei nicht oder nur initial hypoxischen HPV-assoziierten Oropharynxkarzinomen klinisch durchführbar und sicher ist.

## Patienten und Methoden

Patienten mit HPV-assoziiertem Oropharynxkarzinom (*n* = 16) oder zervikalem CUP-Syndrom (*n* = 3) in den Stadien T1–2, N1–2b (7. TNM-Edition) wurden zwischen Juli 2015 und Oktober 2016 in diese monozentrische, prospektive Deeskalationsstudie (NCT00606294) eingeschlossen und erhielten nach Resektion des Primarius, die ohne primäre Neck-Dissektion stattfand, eine prä- und peritherapeutische Hypoxiebildgebung mittels ^18^F‑Fluormisonidazol-PET (FMISO-PET) zur Quantifizierung der Hypoxie der verbliebenen zervikalen Lymphknotenmetastasen. Patienten ohne prätherapeutische Hypoxie oder mit bildgebend detektierter Auflösung der Tumorhypoxie innerhalb der ersten beiden Wochen unter platinbasierter RCT erhielten eine reduzierte Dosis von 30 Gy, wohingegen Patienten mit persistierender Tumorhypoxie einer Standardbehandlung mit 70 Gy zugeführt wurden. Eine Neck-Dissektion 4 Monate nach Ende der RCT diente der Überprüfung des Therapieansprechens.

## Ergebnisse

Während 6 Patienten bereits prätherapeutisch keine Tumorhypoxie ihrer zervikalen Lymphknotenmetastasen aufwiesen, kam es bei weiteren 9 Patienten zu einer frühen Auflösung der Tumorhypoxie, sodass insgesamt 15 der 19 eingeschlossenen Patienten eine reduzierte RCT mit 30 Gy in 15 Fraktionen erhielten. Bei 11 dieser 15 Patienten konnte mittels Neck-Dissektion 4 Monate nach Therapieende eine pathohistologisch bestätigte Komplettremission (pCR) vorgefunden werden. Bei einer medianen Nachbeobachtungszeit von 34 Monaten betrug die lokoregionale Kontrolle nach 2 Jahren 94,4 % für die gesamte Kohorte, und es wurden keine höhergradigen radiogenen Toxizitäten in der Deeskalationsgruppe beobachtet. Mittels Genomsequenzierung wurde ein DNA-Reparatur-Defekt identifiziert, welcher sowohl in der Subgruppe von Patienten mit pCR als auch in einer externen Validierungskohorte signifikant mit dem Ansprechen nach RCT korrelierte.

## Schlussfolgerung der Autoren

Durch einen auf einer Hypoxie-PET basierenden patientenspezifischen Deeskalationsansatz konnte in der vorliegenden, hier diskutierten Studie bei etwa 80 % der Patienten die Radiotherapiedosis um mehr als die Hälfte auf lediglich 30 Gy reduziert werden. Das schlug sich in einer exzellenten Verträglichkeit bei vielversprechenden pCR-Raten nieder. Die Autoren identifizierten und validierten zudem einen DNA-Reparatur-Defekt als prädiktiven Marker für eine pCR nach deeskalierter RCT.

## Kommentar

Wegen der bekanntermaßen besseren Prognose von HPV-positiven Oropharynxkarzinomen werden aktuell in zahlreichen Studien Ansätze zur Deeskalation der Radio(chemo)therapie untersucht. Die Intention hierbei ist, das Risiko einer möglichen Übertherapie für betroffene Patienten zu senken und so eine signifikante Reduktion der behandlungsbedingten Nebenwirkungen zu erreichen. Neben aktuell laufenden Phase-III-Studien für das Gesamtkollektiv der HPV-positiven Oropharynxkarzinome untersuchen die Studiengruppen zunehmend Deeskalationsansätze für bestimmte Subgruppen HPV-positiver Karzinome, beispielsweise nach Therapieansprechen auf eine Induktionschemotherapie [1].

In der vorliegenden 30-ROC-Studie wurde der Primärtumor im Oropharynx reseziert; Patienten mit einem zervikalem CUP-Syndrom erhielten eine Tonsillektomie. Die tumorbefallenen zervikalen Lymphknoten jedoch wurden primär nicht operativ angegangen, sondern erst 4 Monate nach der postoperativen RCT mittels Neck-Dissektion entfernt. Patienten, die entweder vor oder in den ersten beiden Wochen der Strahlentherapie keine intratumorale Hypoxie in den verbliebenen Lymphknotenmetastasen aufwiesen, erhielten lediglich 30 Gy in 15 Fraktionen und konkomitierend eine platinhaltige Chemotherapie [2].

Der Stellenwert der tumorassoziierten Hypoxie für das Ansprechen von Kopf-Hals-Karzinomen auf eine Radiotherapie ist relativ sicher belegt, und die prognostische Relevanz einer frühen Hypoxieauflösung innerhalb der ersten beiden Bestrahlungswochen konnte in mehreren prospektiven Studien demonstriert werden [3–5]. Bereits in den vergangenen Jahren wurde eine auf Hypoxiebildgebung basierende moderate Strahlentherapiedosis-Deeskalation bei Patienten mit HPV-positiven Oropharynxkarzinomen von derselben Arbeitsgruppe untersucht [6]. Hier erhielten diejenigen Patienten mit fehlender initialer Tumorhypoxie oder Auflösung der Hypoxie innerhalb der ersten Behandlungswoche eine Strahlentherapiedosis-Deeskalation um 10 Gy auf die tumorbefallenen Lymphknoten (60 Gy anstatt 70 Gy). Diese moderate Dosisdeeskalation, welche allerdings nur bei 30 % der insgesamt 33 Patienten möglich war, resultierte in einer lokoregionalen Kontrolle und einem Gesamtüberleben von jeweils 100 % nach 2 Jahren; allerdings steht zu vermuten, dass auch bei einer Dosisreduktion um 10 Gy noch in signifikantem Maße radiogene Spätfolgen auftreten können. Daher wurde in der hier besprochenen Pilotstudie bei einem kleinen Patientenkollektiv noch einmal auf 30 Gy weiter deeskaliert. Trotz der nun um mehr als 50 % reduzierten Dosis wiesen 11 von 15 so behandelten Patienten 4 Monate nach Bestrahlung eine pCR auf, 2 weitere zeigten lediglich noch minimale Tumorzellreste mit unklarer Vitalität. Während es bei keinem der Patienten ohne prätherapeutische Tumorhypoxie (*n* = 6) zum pathohistologischen Nachweis vitaler Tumorzellen kam, hatten 4 von 9 Patienten mit initialer Tumorhypoxie trotz Hypoxieansprechen nach 30 Gy noch residuelle Tumorzellen. Möglicherweise ist also ein solcher Deeskalationsansatz besonders für Patienten mit fehlender initialer Tumorhypoxie eine Option. Alle Patienten erhielten trotz Deeskalation der Strahlendosis eine konkomitante platinbasierte Chemotherapie mit 2 Zyklen Cisplatin à 100 mg/m^2^ Körperoberfläche.

Die Ergebnisse dieser Pilotstudie sind vielversprechend: Die deutliche Deeskalation ging mit einem niedrigen Toxizitätsprofil einher: Kein Patient erlitt akute Grad-3–5-Toxizitäten oder musste mit einer PEG versorgt werden. Das bedeutete eine erhebliche Verbesserung im Vergleich zu den bisherigen Deeskalationsstudien [7, 8]. Eine ähnlich dramatische Dosisdeeskalation wurde auch in der MC1273-Studie untersucht: Hier erhielten die Patienten mit HPV-positiven Oropharynxkarzinomen postoperativ 30 Gy oder – im Fall von Kapseldurchbruch bei Lymphknotenmetastasen – 36 Gy in Einzeldosen von 1,5 Gy oder 1,8 Gy zweimal täglich. Bei keinem der 79 in die MC1273-Studie aufgenommenen Patienten waren 6 Monate nach der RCT höhergradige chronische Toxizitäten aufgetreten [9].

Auch hinsichtlich der onkologischen Endpunkte zeigte die vorliegende Studie respektable Ergebnisse: Die lokoregionale Tumorkontrolle lag im Gesamtkollektiv nach 2 Jahren bei 94,4 %, während das progressionsfreie Überleben und Gesamtüberleben zu diesem Zeitpunkt 89,5 % bzw. 94,7 % betrugen. Selbstverständlich muss berücksichtigt werden, dass die verpflichtend durchgeführte Neck-Dissektion 4 Monate nach Ende der Strahlentherapie die Ergebnisse signifikant verbessert haben könnte: Trotz der prinzipiell hohen pCR-Raten könnte die zweizeitige Resektion etwaige noch verbliebene und radioresistente Tumorzellklone eliminiert haben und so trotz der aggressiven Deeskalation zu einer längerfristigen adäquaten lokoregionären Kontrolle beigetragen haben. Aus diesem Grund müssen die vorliegenden Ergebnisse durch die bereits geplante nichtrandomisierte Phase-II-Studie (NCT03323463) und weitere Studien noch bestätigt werden, bei denen auf eine verpflichtende Neck-Dissektion nach der RCT verzichtet wird.

Eine besondere Stärke der Studie ist das begleitende translationale Forschungsprogramm inklusive Genomsequenzierung, RNA-Transkriptom-Analysen und peritherapeutischer MRT-Bildgebung sowie Quantifizierung der zirkulierenden zellfreien HPV-DNA, auch wenn wegen der kleinen Patientenzahl hier die Aussagekraft eingeschränkt ist. Im Gegensatz zu einer kürzlich veröffentlichten Studie, die einen Zusammenhang zwischen der Menge an zirkulierender HPV-DNA und dem Therapieansprechen HPV-positiver Oropharynxkarzinompatienten zeigte, konnte in der vorliegenden Arbeit die Menge an zirkulierender HPV-DNA nicht mit dem Outcome korreliert werden [10]. Um diese diskrepanten Ergebnisse zu erklären und den Stellenwert von HPV-DNA-Messungen im Plasma als potenziellen prädiktiven Biomarker zu verstehen, müssen weitere Studien mit größeren Patientenzahlen abgewartet werden. Dagegen konnten die Autoren aber zeigen, dass das Vorliegen vermehrter Deletionen mit Mikrohomologie als Indikator für einen Defekt in einem zentralen Reparaturweg für DNA-Doppelstrangbrüche mit einer signifikant höheren Wahrscheinlichkeit für das Erreichen einer pCR assoziiert war. Der prädiktive Wert dieses DNA-Reparatur-Defekts für die pCR konnte in einer zweiten externen Kohorte bestehend aus 19 Patienten der MC1273-Studie bestätigt werden. Die Autoren vermuten, dass Zellen mit diesem DNA-Reparatur-Defekt vermehrt Strahlenschäden über einen sehr fehleranfälligen Reparaturweg („microhomology-mediated end-joining“ [MMEJ]) zu beheben versuchen, wodurch die hohe pCR-Rate nach 30 Gy erklärt werden könnte.

Trotz dieser vielversprechenden Ergebnisse sind natürlich die geringe Patientenzahl von 19 Patienten, die noch nicht ausreichend lange Nachbeobachtungszeit von im Median knapp 3 Jahren und der mögliche Bias der verpflichtenden Neck-Dissektion nach RCT zentrale Limitationen, welche die Aussagekraft der Studie einschränken. Außerdem muss berücksichtigt werden, dass die peritherapeutische Erfassung der Hypoxiedynamik mittels FMISO-PET/CT hohe logistische Anforderungen an Patient und Zentrum stellt. Gegebenenfalls lassen sich zukünftig molekulare oder bildgebende Surrogatparameter für die komplexe Hypoxiebildgebung identifizieren [11].

## Fazit

Trotz aller Limitationen bietet diese Studie erste Anhaltspunkte dafür, dass für biologisch selektierte Subgruppen von Patienten mit HPV-positiven Oropharynxkarzinomen eine aggressive Deeskalation der Radiotherapiedosis mit der Konsequenz, höhergradige Toxizitäten zu vermeiden, möglich sein sollte. Das Potenzial eines patientenspezifischen Deeskalationsansatzes für Subgruppen von Patienten mit HPV-assoziierten Oropharynxkarzinomen mittels funktioneller Hypoxiebildgebung ist somit ein wichtiger Schritt in Richtung einer personalisierten Radioonkologie im Kopf-Hals-Bereich. Mit Spannung erwartet werden dürfen die Ergebnisse der Phase-II-Studie, in der der hier verfolgte FMISO-basierte Deeskalationsansatz an einer größeren Kohorte (300 Patienten) überprüft wird. Die Rekrutierung der ersten 150 Patienten, bei denen auf eine initiale Tumorresektion des Primärtumors und die posttherapeutische Neck-Dissektion verzichtet wird, ist bereits abgeschlossen.

*Alexander Rühle und Nils H. Nicolay, Freiburg/Brsg.*
